# Three Decades in Econophysics—From Microscopic Modelling to Macroscopic Complexity and Back

**DOI:** 10.3390/e24020271

**Published:** 2022-02-14

**Authors:** Alex Smolyak, Shlomo Havlin

**Affiliations:** Department of Physics, Bar-Ilan University, Ramat-Gan 52900, Israel; havlin@ophir.ph.biu.ac.il

**Keywords:** econophysics, dynamics of complex networks, cascading failure, network science

## Abstract

We explore recent contributions to research in Econophysics, switching between Macroscopic complexity and microscopic modelling, showing how each leads to the other and detailing the everyday applicability of both approaches and the tools they help develop. Over the past decades, the world underwent several major crises, leading to significant increase in interdependence and, thus, complexity. We show here that from the perspective of network science, these processes become more understandable and, to some extent, also controllable.

## 1. Introduction

Historically, physical science deals with everything that surrounds us, from the smallest to the largest objects of our universe, with the small exception of life, which is mostly explored by biology, and human life specifically, which is handled by psychology, economics and many other sciences trying to find regularities, causalities and in general better understand our daily lives. The general implicit guiding principle of physics, reductionism, impeded physicists researching domains that are (at least to some extent) irreducible. The winds started to change about half a century ago, with the understanding that emergence is an important property in many realistic systems [1], and the mathematical apparatus developed in statistical physics is very useful in modelling and analyzing many everyday phenomena [2,3].

Written in the late 18th century, Adam Smith’s Wealth of Nations [4] is considered the starting point of economic theory. Since then, theory broke into micro and macro, as well as a multitude of schools and approaches, from the simple to extremely complicated, from linear to partial differential equations. From the microscopic modelling perspective, the one looking at asset prices, the prevailing assumption was that prices [5] or price changes [6] follow a Gaussian random walk. This assumption means, on one hand, that the future could not be predicted from the present, and more importantly, risk from movement of assets was easily quantifiable and manageable. The macroscopic view deals with national income, gross domestic product (GDP), employment, production and typically does not concern itself with individual constituents. While it is clear that the macro is made up of the micro, the scales and layers between the individual economic agent or the single change in price of an asset and the contribution of a certain sector to the next year’s GDP make it impossible to deduce one based on the other, or even assess their mutual dependence.

A major difference between physics and economics is the difficulty to set up controlled experiments. In physics, observation of nature will typically lead to hypotheses that could be translated into experiments to test them. In economics, with the possible exception of behavioural economics often tested on college students [7,8], observation is the only possible way to evaluate theory. While the macro-level view provides limited chances to assess accuracy, on the micro level it is to some extent easier. Asset prices, for example, are easily observable, with fine-grained information available and accessible. Those lend themselves to in-depth analysis, if not actual experimentation. Several real-life extreme events, such as Black Monday, a single day in October 1987 that saw the main index of the US stock market plunge over 20%, the collapse of a Nobel laureate backed fund, Long Term Capital Management L.P. in 1998 following several local crises and the economic meltdown following the housing market bubble together with predatory lending and a large web of financial derivatives tied to the housing market, gave experimental evidence to the immense interconnectedness of the micro and the macro, and the severe underestimation of risk by many of the prevalent economic theories.

These two aspects benefited greatly from research conducted by physicists over the last several decades under the common topic of a relatively new field of Econophysics [9,10,11,12]. In this brief review we shortly discuss a sample of several studies that focus on the micro- and macroscopic modelling and analysis to familiarize the interested reader in the usefulness and potential of both perspectives. We wish to highlight the close relationship between the micro and the macro, specifically showing how and where models from physics applied to economic and financial entities bring about the emergent properties that make the field of econophysics so challenging and interesting. From the early insights and models [13], their extensions [14,15] through what has become known as stylized facts [16] of the financial markets, including behaviour of prices and their volatility, and to the inherent connectivity driving global risk [17], tools and methods from physics and complexity sciences [18,19], such as phase transitions [2,3,20], fractal and multifractal analysis [21] and network science [22,23,24,25], all help to understand the intricacies of our economic and financial lives. The following sections will move back and forth between the micro and macro perspective highlighting some recent research driving econophysics forward.

## 2. Macro-Complexity, or the Interconnectedness of All Things

**Figure 1:**
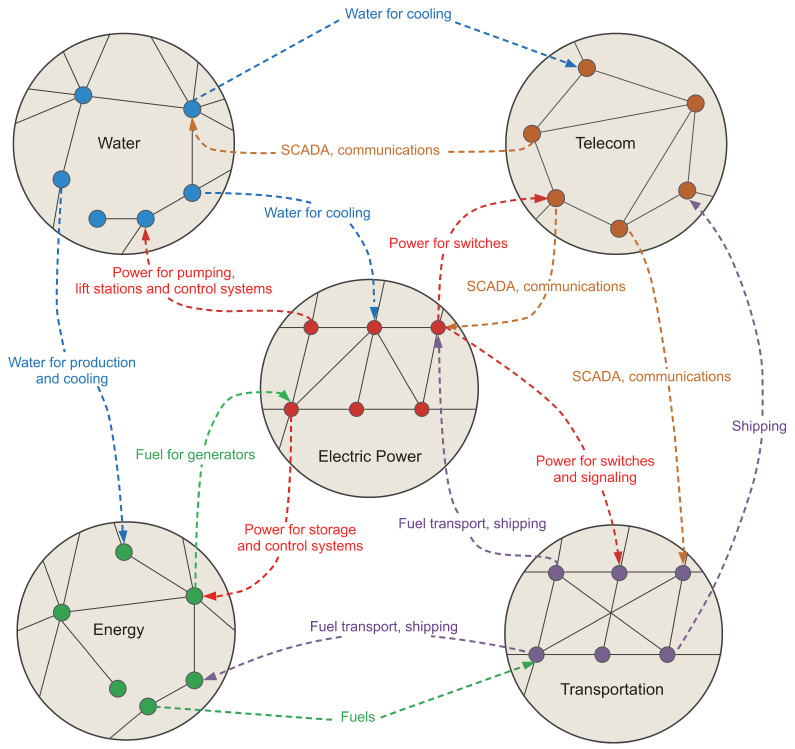
Interdependence in critical infrastructure. This schematic, after [50], shows the rich dependency coupling between different networks. Each circle is a complex network in its own right, with its degree distribution, connectivity and potential for random or intentional failure. These dynamics are greatly exacerbated due to the introduction of inter-network dependence. Each of the networks described requires some critical resource from one or more of the other networks to operate smoothly with failure in networks providing said critical resources may propagate failures to other networks.

The physical infrastructure that surrounds us is a good starting point for the macro to micro journey and back. While it may not seem obvious, interdependence in infrastructure is critical to its continuous operations and resilience [26,27]. Figure 1 shows a schematic of various dependencies between different elements of such infrastructure. Those may be immediate (water, electric power) or longer-term (fuel, long-range transportation) but it is clear that efficient operation of every element depends either directly or indirectly on every other element. A prototypical example of interdependent network and connected infrastructures exhibit non-trivial and seemingly unpredictable transitions from operational to failed [28,29], unexpected critical junctions and positive, as well as negative, feedback loops.

Various theoretical models have been developed to analyze the resilience and onset of failure on such networks over the past decade or so [23,30,31,32,33,34,35,36,37,38,39,40,41,42,43,44,45], shedding light on the importance of degree distributions, intra- and inter-layer connectivity and mitigation strategies. We note here this list is far from exhaustive and focuses mainly on contributions from network science. Extensive literature exists from an economics perspective, and we refer to publications such as [46,47,48] and references therein as important examples of a complimentary view. As with the system under investigation, those models often take a probabilistic, generating function approach as their starting point. In its most fundamental form, a degree generating function is defined as $G_{0}\left(x\right)=\sum_{k=0}^{\infty }p_{k}x^{k}$ [49], where taking the $k^{th}$ derivative and equating *x* to zero gives the probability of having a degree of *k*. Taking this idea further, various connectivity constraints can be built into the generating functions and probabilities can be calculated to represent objects of interest, such as a surviving component size, given a defined connectivity and initial failure. Thus, if we have an initial model of how our networks are set up and connected, and we can specify how they fail, we can determine what they will look like when the process of failure plays out.

These models are very powerful in the sense that they allow us to predict the infinite-time states of systems under cascading failures in the presence of complex interactions. The solution of the model showed that, due to dependencies, a microscopic failure of a single node can yield a macroscopic cascade and an abrupt collapse of the system [51,52]. They do suffer from some drawbacks, however. From the technical perspective, generating functions become very cumbersome for non-trivial or, worse yet, empirical distributions. We can solve them for simple cases such as regular, random (Erdos-Renyi, ER) networks, and for scale free (SF, power-law) distributed ones. However, we know these mathematically convenient constructs do not represent real networks. For example, they can not consider spatial embedding constraints. Nevertheless, it was shown by Bashan et al., both analytically and via simulations, that spatially embedded interdependent networks are far more vulnerable to microscopic failures [52]. Moreover, a recent analytical study that considered spatiality and cascading failures in modular interdependent networks was carried out by Vaknin et al. [53]. Lastly, because they deal with probabilities over the entire system, these macroscopic models have a hard time dealing with specificities such as individual nodes or small systems. When looking for answers at the level of the individual power plant or internet hub, macroscopic models are less helpful.

## 3. Microscopic Failure and Recovery Models—From the Lab to the Exchanges

To gain better insight into the behaviour of our system, more specifically, to enable analysis of its dynamical properties in addition to the long-term state, we turn to specifying individual node dynamics, which may include probabilities for failure [54] as well as recovery depending on its internal state and the state of its neighbours [55,56]. The methods described in the previous section are still useful and allow us to calculate steady state solutions for various parameters and detect non-trivial transitions between those states, but only when we switch our view to a microscopic one, and let a simulated system run its course can we discover the rich dynamics. Importantly, analytical solutions typically assume infinite time and size, while in life the actual time scales may be very important and systems are more often than not small in thermodynamic terms.

**Figure 2:**
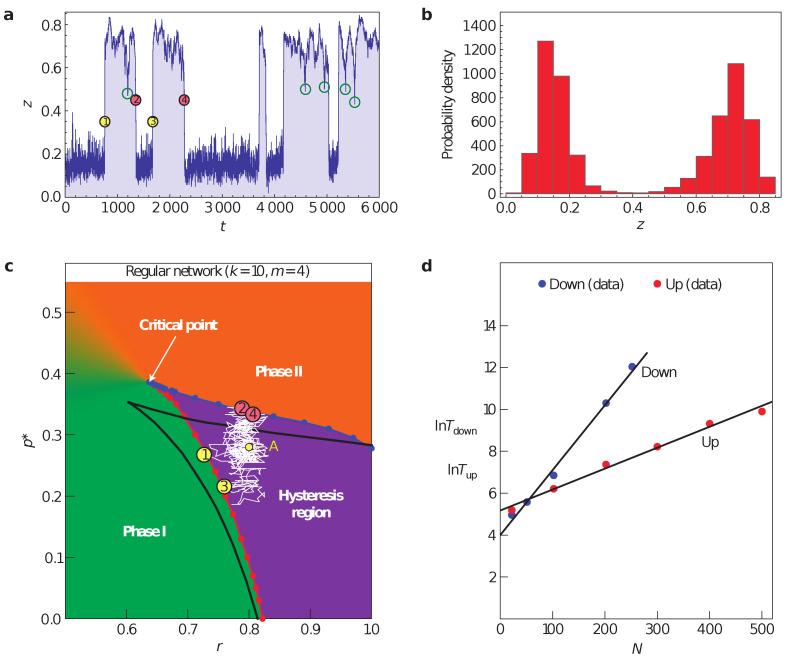
The importance of microscopic modelling. A simulation of a model with spontaneous recovery from failure on a regular network with k = 10 made of 100 nodes. (**a**) A realization of the time evolution starting from point A in panel (**c**) with switching between the failed and active states marked by yellow circles, and failure to complete a transition by green circles along the time series. (**b**) The probability density function of (**a**). (**c**) The phase space for the specified system, with the while line showing the path realized in (**a**), yellow and red circles matching the above. (**d**) The expected life time of the system in each state given a network size. After [55].

In particular, we may specify our model such that nodes in our network may fail spontaneously, fail under the influence of their neighbours or, if they are in a failed state, spontaneously recover with a certain probability density over a time period. As shown in Figure 2, a system under a simple failure-recovery model behaves highly non-trivially over time, not only spending long periods of time in its “active” or “failed” states, but also exploring transitions from one to the other without completing them due to the hysteresis region. As discussed in [55], this behaviour and the resulting bi-modal distribution of the system is very similar to the observed in financial markets when evaluating the fraction of companies listed in various indices with positive vs. negative returns.

**Figure 3:**
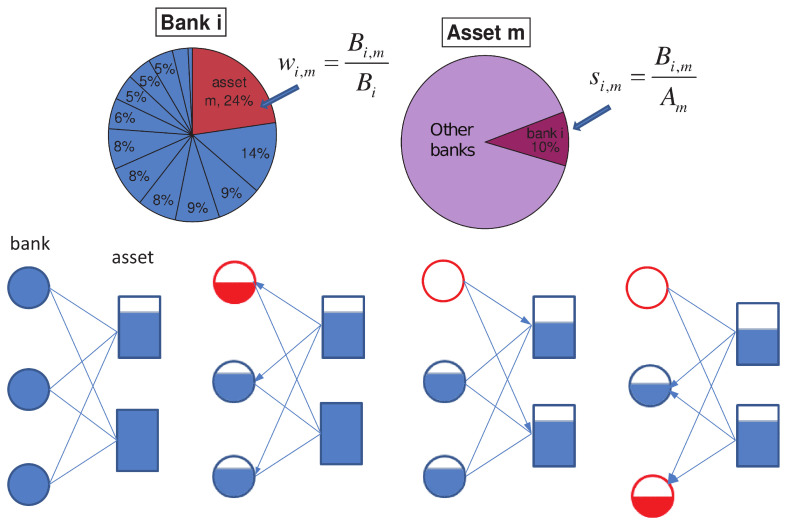
A microscopic model of a bank–asset risk propagation model. The relatively simple and intuitive model is very instructive and facilitates further modelling and analysis. The Bank’s holdings are distributed between multiple assets and the assets are held by various banks. An initial reduction in the value of an asset leads to an impact to the values of all the banks exposed to it. For some banks the exposure is large enough to cause the bank to fail leading to further sell-off of its assets inducing a potential cascade. After [57].

Temporal dynamics, small scales and fluctuations around the analytical solutions all highlight the importance and practical use of microscopic modelling. An instructive example was developed in Ref. [57]. The model specifies a risk-propagation mechanism on a bipartite Bank-Asset network, Figure 3. A bipartite network of banks and the assets on their books is set up, along with several parameters governing initial shock and levels of propagation at a node level. Then, an initial shock to an asset results in loss of value for a bank holding that asset. Enough such shocks may lead to a failure of a bank, leading to subsequent devaluation of the assets on its books. Importantly, the simple model allows one to build back up from the micro to the macro. In Ref. [57], the authors analyze the sensitivity of the whole system to various loan classes showing close relations with the events of real life. Below we discuss how such models can be taken a step further.

## 4. Failure and Immunization in Real, Macroscopic Networks

The microscopic model presented in the previous section gives us powerful tools to stress-test our system against various potential failures and estimate their relative importance. There is, however, more to be done. As discussed in Ref. [58], given a specification of the failure process, the microscopic model can reveal nodes in the network that, more than others, propagate failure. These nodes do not display any high centrality values, and yet ensuring their protection from failure helps keep the network intact relatively cheaply in terms of number of nodes protected. Importantly, those nodes can be identified with only knowledge of their local neighborhood, without complete information of the entire network. Partial knowledge does not interfere with the performance of the suggested method. All that is needed is knowledge of the failure mechanism in order to devise an efficient mitigation strategy.

**Figure 4:**
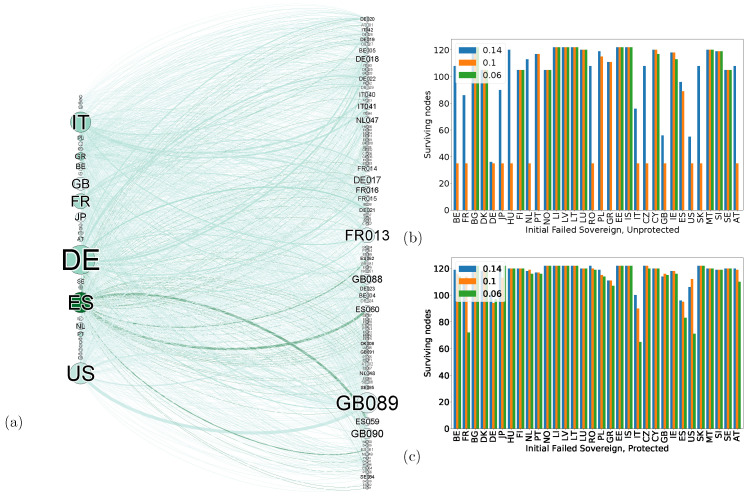
A macroscopic experiment showing the structure and cascading failure dynamics of a real Bank-Asset network. (**a**) The bipartite network, left part are the sovereign assets, right are the holding banks. Node size reflects the capital (either held or invested), edge colour shows size of holding, the darker the larger. (**b**) Survival of the unprotected network. The x-axis shows the initial failing sovereign debt asset, while the y-axis shows the number of remaining nodes. The colours show different levels of sensitivity. The higher the sensitivity, the bigger the impact needed to cause a failure. With the exception of very small countries, most failing sovereign debts cause a cascading failure of the entire network, some even for relatively resilient conditions. (**c**) The same network as (**b**) but with the defense mechanism in place, even the most sensitive networks do not undergo a complete cascading failure with the protection, leading to a much more stable overall economic environment. After [58].

It is now possible to expand the local node-level insights up to a network-level view. Both in simulations and, more importantly, in real networks, we can use the microscopic model for macroscopic benefit. Figure 4 shows just that. The left panel visualizes a network of banks (right) and sovereign debt (left). The top right panel shows the fragility of the network under the default of various states given a certain threshold (colour bars). Most countries failing lead to cascading failure across the entire system for the lowest threshold, and many cause significant damage even with a high one. Protecting the nodes, the microscopic model highlights enable the entire network to remain mostly intact under most failing conditions.

Extending the model to other networks, failure mechanisms and topologies allow to protect many types of networks, bipartite or otherwise, from cascading failure stemming from unknown source. That feature is very important due to the inherent unknowability of every source of risk. Once the mitigation strategy is able to not care where the cascade starts, we know it will serve us well no matter the manifestation of risk. Mitigation and recovery models discussed here offer possible paths to alleviation of systemic financial risk. Those models highlight vulnerabilities and potential protection or recovery methods but are not yet applied (to our knowledge) in decision making by regulators. Thus, the practical ability of such approaches to positively impact economics is yet unknown.

The networks discussed here are created from exposure between various entities. Econophysics allows us to explore and analyze networks whose impact on the economy goes beyond the financial. Next, we explore another network constructed from the micro that gives tools to understand the economy as a whole. For that, we turn to mobility networks.

## 5. Micro-Mobility to Macroeconomics—From Cell Phones to GDP Estimates during a Pandemic

The year 2008 taught us important lessons about risk management, and made us understand that diversification, previously thought to be a strong risk-immunization technique, can fail catastrophically. We found out to which extent various financial and economic entities were interconnected, how that connectivity was often hidden from sight and how extreme its ramifications can be. Excess greed in the lending market for houses in the United States led to a credit crunch that almost toppled the entire global economic system. The abyss was avoided only by massive government bail-out plans, echoes of which are still with us today, more than a decade later. The year 2019 brought about a very different type of stress factor—this time a pandemic, a virus first recorded in Wuhan, China, and quickly spread throughout the globe. Response varied greatly between different countries, with many different non-pharmaceutical interventions (NPIs) taking place. Some pursued tight lockdown measures while others refrained from implementing strict limitations, striving for social distancing and herd immunity. Estimating the success of these measures is beyond the scope of this review, but the measures taken had indubitable consequences from an economic perspective. Global and local limitations on mobility led multi-national companies as well as individuals to find themselves disconnected, without air travel or public transportation, and with most businesses closed.

In recent years, proliferation of cellular devices with accurate GPS sensors, coupled with multiple companies that gather and process the data, exposed large amounts of fine-grained mobility data to researchers [59]. Several directions of research explored effects of lockdown and similar restrictions through the perspective of mobility networks [60,61,62,63,64]. Many of those deal with the effects of restrictions on epidemic spreading and vice versa, but some focus on the economic and social ramifications of the restrictions.

**Figure 5:**
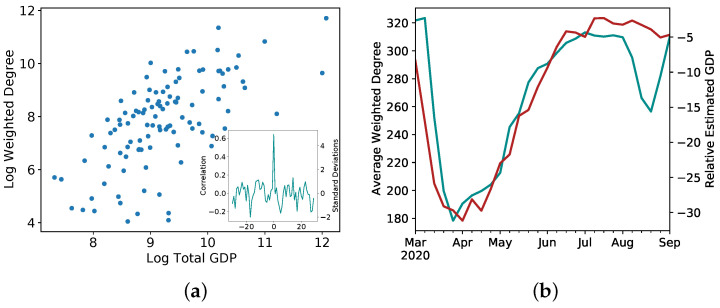

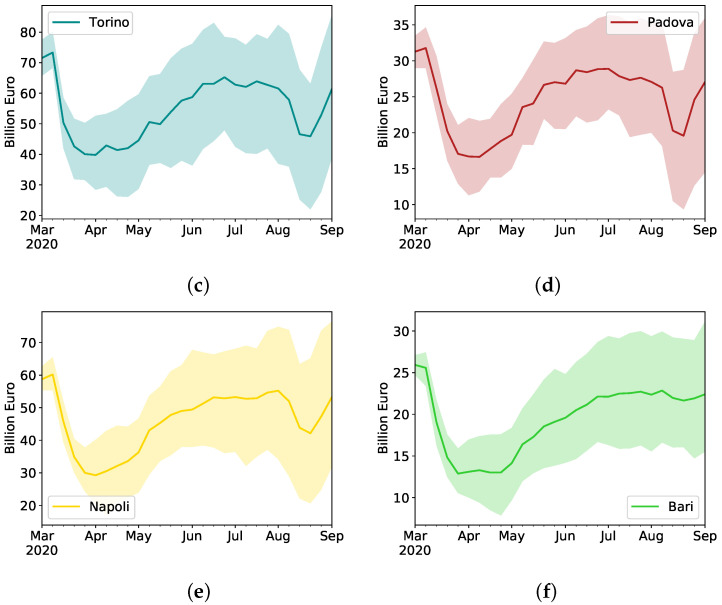
From micro to macro, inferring the economy from mobility, after [63]. (**a**) The correlation between the GDP levels of individual Italian provinces for 2017 and the weighted province degree. Log of values is shown to highlight the relation holds regardless the strongest province economies. Inset: shifted correlation to validate the significance of the relationship. (**b**) The Average weighted degree, teal, left axis; and the estimated real-time GDP, red, right axis. (**c**–**f**) The Province-based GDP forecast based on mobility data for Turin, Padua, Como and Pisa Provinces. The thick line is the average forecast, with the shaded area showing the 25–75 percentile range.

In Ref. [63], the authors start from the microscopic mobility patterns of individuals and construct a country-wide network of mobility in Italy, before, during and after the first lockdown. Various parameters of the emerging networks are analyzed, from the perspectives of scaling, dynamics and resilience. Interestingly, strong relations are found with two major economic indicators. Statically, i.e., using a historical point in time, regional mobility levels correlate strongly with official levels of regional GDP. Further, using a fast-moving estimate of the GDP based on several measures (and on its own shown to match the actual GDP when available), they show how well changes in levels of mobility correspond to changes of GDP, and from that they derive the local (regional) estimates of GDP (Figure 5), in near-real time, by far faster than official calculations. This estimate, easily calculated from available data, could allow decision makers to gauge the magnitude of economic impact to various regions following planned restrictions. Thus, again, a microscopic model gives macroscopic visibility into system behaviour and stability.

## 6. Trading, Failure and Centrality—From Local Thresholds to Global Importance

**Figure 6:**
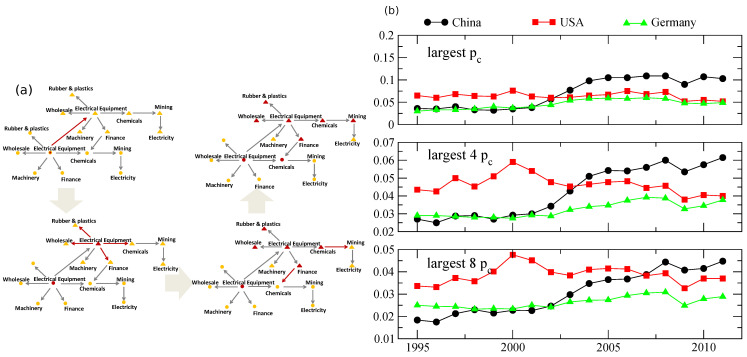
Tying things together, after [65]. (**a**) The propagation of cascading failure over a network of interacting industries and countries. Triangles and circles are different countries with interaction between and within countries facilitating the cascade. (**b**) A critical threshold exists beyond which the network fails. The higher the threshold, the more sensitive the network is to that failure. Looking at the top 1, 4 and 8 such critical thresholds a pattern emerges whereby Chinese sectors take a more central position in the networks.

Finally, we bring together some of the methodologies described before to compare different countries and sectors and their importance to the global economy. The authors in Ref. [65] apply a model very similar to the one described in Section 3 after [57], only instead of banks and assets the network is comprised of various industries trading with each other. They then use sensitivity of the network to failure of a network’s constituent to assess its relative importance. That is, similarly to the threshold shown in Figure 4, the higher the threshold above which the network undergoes cascading failure, the more important a sector or a country is. Figure 6a shows the progression of failure in the described network in a different setting than a bank–asset network. Figure 6b shows the results of the analysis. Surprisingly, through the lens of sensitivity to failure and examining the top one through eight sectors, the main drivers of economic activities that historically were in the United States have shifted to other geographies, with China taking the lead for the top component nearly two decades ago and surpassing in all eight top components around 2010.

It is noteworthy to highlight the contrast between the approach taken in [58] as mentioned in Section 4 and the one presented in [65]. While both approaches center around the onset of cascading failure given a specific failure mechanism, the next step is almost exactly opposite. The former approach designs a defense mechanism that, when functioning properly, is agnostic the specifics of the failure’s origin. The latter, on the other hand, uses exactly that magnitude of impact to determine relative importance. Both models can be expanded and refined, each on its own, but the unifying theme, tying together different sections discussed above, highlights the strength of micro- and macroscopic modelling in the application of network science and econophysics for analysis of actual economic systems, their strengths and weaknesses.

## 7. Discussion

Throughout this paper we reviewed several research contributions over the last few years showcasing the immediate applicability of modelling and simulation methods expanded from statistical physics and network theory to various aspects of economics, finance and everyday life. From the big picture is system-level appreciation of the interdependence and nontrivial relations that are present in our most fundamental infrastructure, through fine-grained, local models of interaction that mirror high frequency trading asset behaviour to estimation of local and global economic behaviour in normal and highly anomalous times. These, and many other contributions, give us both new perspectives on known phenomena and mitigation tools in order to manage them such that they do not lead to massive damage. Even with this wide range of topics covered and approaches demonstrated, this is but a grain in the wide landscape of research conducted over the past decades, and evolving still, bridging the gap between methods and discoveries from physics to social and economic life.

Our economy, a textbook case of complex, adaptive system, is comprised of multiple time scales, types of interactions and agents driven by fear, greed, hope and desire, aiming to improve their state compared to themselves and others, poses a great challenge not typically encountered in physics, where particles’ knowledge of governing rules does not change their behaviour. Yet, many patterns emerge over time that show that, nonetheless, there are regularities and laws governing some of the observable outcomes. There are arguably many more open questions, from big to small, yet to be answered pertaining to everyday life in its various aspects (economics, epidemiology, mobility, transportation and many more) compared to“classical” physics, and the growing body of research is a strong corroboration of that. The development of network science, econophysics and sociophysics has laid the directions, but the journey is far from over.
